# Fertility Management in Pollinators: Queen Storage, Transport, and Reproductive Resilience in *Apis mellifera* Under Commercial and Environmental Stressors

**DOI:** 10.3390/insects17060557

**Published:** 2026-05-28

**Authors:** Zunair Ahsan, Faouzi Haouala, Usama Abdullah, Umar Sajid Kayani, Mokhtar Rejili

**Affiliations:** 1 College of Animal Science and Technology, Yangzhou University, 88 South University Rd., Yangzhou 225009, China; mh24057@stu.yzu.edu.cn (Z.A.); usama.kpr124@gmail.com (U.A.); umarkiyani7171@gmail.com (U.S.K.); 2 Department of Biology, College of Sciences, Imam Mohammad Ibn Saud Islamic University (IMSIU), Riyadh 11623, Saudi Arabia; fmhaouala@imamu.edu.sa

**Keywords:** honey bee queen, queen fertility, reproductive resilience, sperm storage, commercial beekeeping, stress physiology

## Abstract

As the only reproductive female in the colony, the honey bee queen (*Apis mellifera*) is essential to colony survival, productivity, and the sustainability of pollination services that underpin food security and global agriculture. The biological, physiological, molecular, and commercial elements that affect queen fertility throughout the commercial lifecycle, from mating and development to sperm storage, banking, transportation, and colony establishment, are examined in this review. According to available data, successful queen reproduction depends on effective mating, long-term sperm viability in the spermatheca, stable hormonal regulation, and adequate nutritional and environmental support. However, a number of interrelated stressors, including temperature changes during storage and transportation, confinement, inadequate nutrition, pesticides, pathogens, parasites, and climate-related pressures, can reduce sperm viability, impair ovarian function, and increase colony losses. Precision apiculture, cryopreservation, instrumental insemination, and omics-based biomarkers are examples of emerging technologies that offer promising techniques to enhance queen resilience and commercial management. However, there are still significant information gaps, especially in the areas of integrated multi-omics techniques across the commercial lifespan and standardized queen-quality evaluation. Future advancements are needed to preserve queen fertility and protect pollination services. This will require integrating reproductive physiology, biotechnology, and commercial management to build climate-resilient, biosecure, and sustainable systems.

## Simple Summary

The world’s food crops depend on honey bees for pollination. Every honey bee colony revolves around the queen, whose ability to produce large numbers of high-quality fertilized eggs determines colony health and survival. A healthy queen must consistently produce fertilized eggs, store sperm for several years, and mate successfully with several male bees (drones). Queen fertility, however, may be harmed by contemporary beekeeping methods, including mass rearing of queens, storing them, and transporting them across great distances. Weak colonies or even colony death result from many queens arriving at their new hives with damaged sperm or diminished egg-laying capacity, resulting in weak or collapsing colonies, economic losses for beekeepers, and reduced pollination services. Queen health is further threatened by stressors such as high shipping temperatures, inadequate nourishment, exposure to pesticides, diseases, and parasites. This paper outlines the main risks queens encounter during their commercial trip from breeder to beekeeper and describes how queen fertility functions at a biological level. It also covers novel technologies that could help preserve queen fertility, such as genetic selection, electronic hive monitoring, and cold storage of drone semen. In addition to being beneficial for beekeepers, understanding and enhancing queen health is crucial for the future of sustainable agriculture and world food security.

## 1. Introduction

### 1.1. Importance of Pollinators

**Figure 1:**
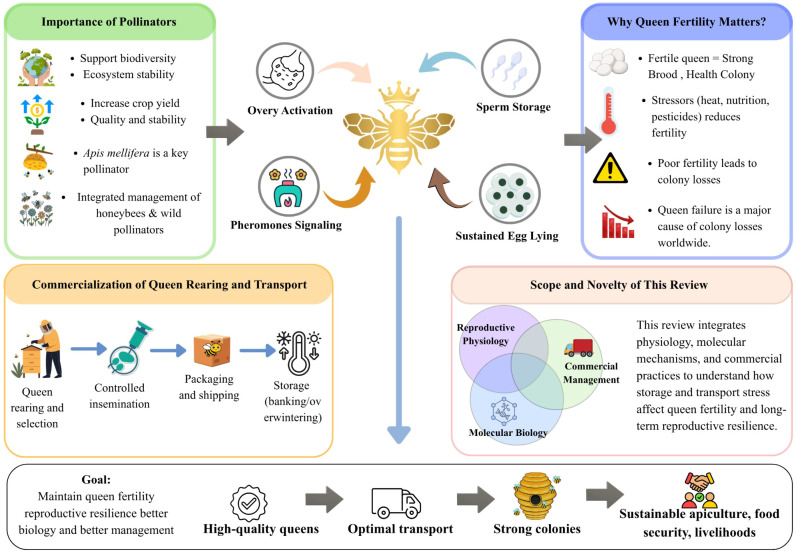
Integrated framework of queen fertility and colony sustainability in *Apis mellifera*. The diagram shows the role of pollinators in crop productivity, food security, and ecological stability. It illustrates how queen processes such as ovary activation, sperm storage, pheromone signaling, and egg laying regulate colony function, while reduced fertility leads to colony decline. It also includes commercial practices (rearing, mating, transport, and storage) and associated stressors affecting reproductive quality. Overall, the figure links reproductive physiology, molecular mechanisms, and management practices to explain colony resilience and pollination success.

Pollinators are essential to both natural ecosystems and agricultural production. They maintain the sexual reproduction of wild flowering plants and improve the quantity, quality, and stability of many cultivated crops (Figure 1). Losses in wild and managed pollinators can reduce pollination services, affecting ecology, the economy, and food security [1]. Pollinators also support farmer livelihoods, beekeeper revenue, agricultural productivity, biodiversity maintenance, and overall human well-being [1]. The benefits of pollination in agricultural landscapes go beyond simply boosting productivity; it also enhances commercial characteristics that affect marketability.

### 1.2. Why Queen Fertility Matters

Queen fertility is a key factor in determining colony productivity, stability, and long-term survival in Apis mellifera colonies (Figure 1). The queen is the primary reproductive individual, responsible for laying fertilized eggs and maintaining colony cohesion through pheromonal signaling. Brood production, worker population size, foraging force, colony growth, and eventually honey production and pollination efficiency are all strongly impacted by a queen’s ability to reproduce. Considerable variation exists in the physical, insemination, and reproductive quality of commercially produced queens, including differences in sperm storage capacity and mating number, all of which are closely associated with queen fitness and colony performance [2]. Studies in Africanized honey bees have shown that heavier queens tend to exhibit higher fecundity, improved colony development, and greater longevity than lighter queens, suggesting that body weight at emergence may serve as an indicator of reproductive quality [3]. However, these findings may not be universally applicable across all honey bee populations and subspecies.

Successful mating, sufficient sperm acquisition, and the long-term preservation of viable sperm in the spermatheca are all necessary for a queen’s reproductive quality. Honey bee queens must store enough healthy sperm to fertilize eggs throughout their lives, yet they only mate briefly in the early stages of their lives. Physiological, behavioral, and molecular changes induced by mating play a critical role in regulating ovary activation, pheromone production, and overall reproductive health in queens [4]. Ref. [5] discovered a high correlation between colony failure and low sperm viability, with queens from failing colonies frequently displaying sperm viability close to 50% as opposed to roughly 85–92% in healthy colonies. In addition to decreasing egg production, low queen fertility can cause the colony as a whole to become unstable. Reduced worker retinue behavior, poor brood care, more supersedure attempts, and ultimately colony loss are all consequences of queens with poor reproductive quality producing weaker pheromonal signals. Reduced colony output and elevated mortality risk are commonly linked to poor queen quality and early queen replacement [6].

Poor brood patterns, low egg production, drone-laying, supersedure, or queen loss are all signs of queen failure, which is a major cause of colony loss. Queen longevity has declined in recent years, with more than 50% of queens in the United States being replaced within six months, despite queens previously surviving for one to two years. This increase in replacement rates has been associated with periods of elevated colony mortality. Several factors contribute to queen failure, including poor mating quality, low sperm viability, disease, nutritional stress, pesticide exposure, and temperature stress [5].

### 1.3. Commercialization of Queen Rearing and Transport

From a local practice, queen production in *Apis mellifera* has developed into a specialized, standardized enterprise that includes shipping, quality assessment, controlled rearing, and mating.

Because of this commercialization, the production and trade of queens have expanded, and selective mating is now a crucial component of breeding initiatives. Ref. [7] highlighted that the movement of queens and other live bee materials continues to be a significant operational aspect of contemporary apiculture because it supports replacement, stock improvement, and pollination-dependent industries, despite the biological and regulatory risks involved.

In areas where periodic colony losses create an early demand for mated queens that local production cannot always supply, international and long-distance queen commerce has become particularly significant. However, the quality of queens that enter these markets varies. Commercial exchange may not always ensure consistent reproductive quality, according to [2], who discovered notable variations in physical characteristics, stored sperm counts, and mating quality among commercially produced queens.

Due to its ability to precisely control paternity and facilitate the transmission of specific features that are challenging to sustain under open mating, instrumental insemination has emerged as a crucial tool in commercial queen breeding. Several honey bee viruses, such as DWV, ABPV, BQCV, SBV, and FV, can be found in semen used for instrumental insemination, according to [8]. This indicates that artificial insemination methods must also be taken into account in connection with pathogen transmission.

Effective shipping techniques are crucial to preserving fertility during transportation since commercial queen production relies on the regular transfer of live queens between breeders and beekeepers. Tracking queen shipments from the United States to Canada, researchers found that queens were exposed to fluctuating transit temperatures, including extreme hot and cold conditions that significantly reduced sperm viability. The study also demonstrated that shipment box design and the presence of attendant bees improved thermal buffering, indicating that packaging conditions directly influence the queen’s reproductive integrity [9].

Ref. [10] showed that indoor overwintering of banked queens can be a viable alternative to imports. Survival rates were affected by bank density. After storage, queens retained sperm viability and counts similar to those overwintered in colonies. However, early oviposition was briefly reduced. Similarly, Ref. [11] discovered that, while overwintered banked queens functioned similarly to recently imported queens in terms of colony size, honey productivity, and winter survival following introduction, they had low overall survival and decreased sperm viability during storage. According to these studies, commercial supply chains can be supported by storage systems, but their effectiveness depends on increasing survival and reducing reproductive degradation while in storage.

### 1.4. Scope and Novelty of This Review

The available literature on honeybee queen biology, reproductive physiology, and commercial queen management is still dispersed across various disciplines, with the majority of studies focusing on specific aspects of queen health rather than integrating the entire continuum from production to long-term reproductive performance (Figure 1).

The current review is unique in that it combines molecular biology, reproductive physiology, and commercial management into a unified framework to explain the establishment, maintenance, and compromise of queen fertility throughout the commercial lifecycle.

### 1.5. Literature Search Strategy

A systematic literature search was conducted to find and compile the most recent data on honey bee queen fertility and reproductive resilience. The databases PubMed, Scopus, Web of Science, and Google Scholar were used to gather the literature. Combinations of “Apis mellifera queen fertility,” “queen reproductive biology,” “spermatheca,” “sperm viability,” “queen banking,” “queen transport stress,” “thermal stress,” “pesticide exposure,” “queen health,” and “commercial beekeeping” were among the search phrases used. The focus was on current studies that addressed reproductive physiology, molecular regulation, environmental stresses, commercial queen management, and developing reproductive technologies. These studies were mostly published between 2000 and 2025. Included were peer-reviewed research on the biology of queen reproduction, sperm storage, mating biology, transport stress, banking, environmental stresses, and reproductive resilience. Excluded were studies that were solely concerned with worker bees and had no bearing on reproduction; non-English publications; and studies that had no direct bearing on queen fertility. The selection of publications was based on title, abstract, methodological significance, and relevance to queen reproductive health and commercial management. Additional relevant studies were identified through citation tracking of key references.

## 2. Queen Reproductive Biology and Fertility Determinants

### 2.1. Queen Development and Mating Biology

The developmental environment, rearing circumstances, and larval nutrition all have a significant impact on the quality of the queen upon emergence. Developmental conditions significantly influence future reproductive performance. Larger queens at emergence show higher fecundity, better colony growth, and longer lifespans than smaller queens [3]. Before beginning mating flights, virgin queens go through a maturation period after emerging, usually during the first week of adulthood. Reproductive tissues develop during this time, and queens become ready for mating both physically and behaviorally. Ref. [12] showed that mating causes significant genetic alterations in the brain and ovaries of the queen, which have an impact on the physiological and behavioral processes linked to the shift from virgin to reproductively active queen state. Honey bee queens are extremely polyandrous and mate with multiple drones during one or more brief early-life mating flights. Ref. [13] discovered that rainfall had a negative correlation with polyandry in native populations of *Apis mellifera jemenitica*, indicating that climatic conditions have a major impact on queen mating frequency. Queens gather semen during mating and store it in the spermatheca for later use. Only a small portion of the millions of sperm that are delivered during copulation are preserved and continue to be viable for years.

### 2.2. Spermatheca Structure and Function

The spermatheca, a specialized sperm-storage organ in *Apis mellifera* that consists of a reservoir, glandular tissue, tracheation, and a duct connecting to the reproductive canal, is essential to the queen’s lengthy reproductive life. Ref. [14] reported via scanning electron microscopy that sperm cells fill the entire volume of the spermathecal reservoir, with some of them situated centrally in the lumen and others in touch with the epithelium. The spermatheca’s job is to keep sperm viable while reducing oxidative and metabolic damage over time. Ref. [15] showed that oxygen levels in the spermatheca are extremely low and that stored sperm in honey bee queens mostly depend on non-aerobic energy metabolism. They discovered that stored sperm had higher expression of glyceraldehyde-3-phosphate dehydrogenase than ejaculated sperm, and they identified glyceraldehyde-3-phosphate as a crucial substrate for sperm survival. These results suggest that the spermatheca offers a low-oxygen environment that promotes energy-efficient sperm maintenance and inhibits oxidative stress. Additionally, molecular data suggests that the spermatheca and its secretions actively contribute to the long-term maintenance of sperm. Ref. [16] demonstrated that, compared to virgin queens, mated queens express more antioxidant genes in the spermatheca, such as catalase, thioredoxin 2, and thioredoxin reductase 1. This increase suggests that one important mechanism shielding stored sperm from reactive oxygen species is antioxidant defense. Ref. [17] identified transcriptomic changes between virgin and mated queen spermathecae, including altered expression of genes involved in sperm storage, carbohydrate transport, and metabolism. These findings suggest that regulated molecular activity in spermathecal cells is essential for sperm preservation and reproductive efficiency throughout the queen’s life.

### 2.3. Long-Term Sperm Maintenance and Molecular Regulation of Fertility

While [18] found metabolic pathways that probably contribute to the biochemical protection of stored sperm, Ref. [15] directly linked low oxygen circumstances and modified metabolic pathways to the avoidance of reactive oxygen species damage. Ref. [19] discovered that lysozyme, a conserved immunological effector, was one of the spermathecal fluid proteins linked to decreased sperm viability. They interpreted this negative correlation as proof of a trade-off between immunity and sperm maintenance. Collectively, these studies show that low-oxygen metabolism, antioxidant buffering, and carefully controlled immunological activity within the spermatheca are all necessary for long-term sperm survival. A coordinated network of hormones and molecules controls the fertility of honey bee queens. One important regulator of queen fertility and reproductive differentiation is juvenile hormone. Juvenile hormone directly improves reproductive capacity throughout ontogeny, as demonstrated by [20], who demonstrated that juvenile hormone supplementation during larval development boosted ovariole number and enhanced hemolymph vitellogenin levels in in vitro reared queens.

### 2.4. Integrated Biomarkers of Reproductive Health

A collection of morphometric and phenotypic characteristics that represent developmental circumstances and reproductive capacity can be used to evaluate queen quality. Because they are directly related to queen fertility, mating capacity, and colony performance, body weight, thorax breadth, and ovariole quantity are the most commonly utilized markers among these.

Although ovariole quantity differed widely among commercial queen sources, Ref. [21] found that in most samples, it did not exhibit an overall association with wet weight or numerous other exterior features, suggesting that body weight alone is informative but insufficient. Because it indicates structural development and has been used in conjunction with body weight to assess queen quality, thorax width is another significant external factor. Since ovariole quantity establishes the structural foundation for egg production, it is a direct internal indicator of reproductive capability. In comparison to queens raised from emergency cells or older larvae, Ref. [22] discovered that queens raised from one-day-old larvae had the largest ovariole counts, along with superior spermathecal diameter and ovary features. Ref. [23] discovered that while intense mass raising resulted in smaller queens with decreased thorax width and body weight, ovariole number and sealed brood area were unaltered, indicating that some external size reductions do not always translate into decreased fecundity. According to [19] sperm viability in queens was linked to particular spermathecal fluid proteins, and lysozyme, a conserved immune protein, had a negative correlation with sperm viability. These findings suggest that oxidative and immune balance are directly related to the quality of queen reproduction. Queen fertility is accompanied by widespread gene-expression reprogramming throughout reproductive and metabolic organs. According to transcriptome research, genes related to sperm storage, glucose metabolism, and tissue-specific reproductive activity are expressed differently in the spermathecae of mated queens than in virgin queens [17]. Using quantitative proteomics of spermathecal fluid, Ref. [19] showed that a small number of proteins, such as odorant-binding protein 14, lysozyme, serpin 88Ea, artichoke, and heat-shock protein 10, are linked to sperm viability. Proteomic profiles may be able to differentiate successful queens from unsuccessful queens based on the negative correlation between lysozyme and sperm survival. Ref. [24] demonstrated that limiting egg laying for 21 days drastically affected queen gut microbiota, decreased ovarian activity, and altered ovarian metabolic profiles, providing direct evidence that queen microbiota is connected with reproductive metabolism. Recently, Ref. [25] demonstrated a substantial correlation between queen quality and host–microbial interactions.

## 3. Environmental and Commercial Stressors Affecting Reproductive Resilience

### 3.1. Thermal Stress

Because stored sperm within the spermatheca is extremely sensitive to temperature changes, thermal stress is one of the most often documented risks to the reproductive resilience of honey bee queens. Even when queens appear to be alive and behave normally, numerous studies have shown that exposure to temperatures below 8 °C or above 40 °C dramatically decreases sperm viability [5]. While queens themselves frequently showed few apparent phenotypic changes, experimental exposure to both heat and cold stress resulted in significant drops in stored sperm survival [9]. In a similar vein, Ref. [19] found that sperm viability significantly decreased outside of a comparatively small thermal safety range, with exposure to 42 °C resulting in significant reductions in fertility. Further research on transportation revealed that temperature spikes associated with shipments and insufficient thermal buffering during transit directly cause damage to queen reproduction [5,9]. All of these results suggest that long-term queen fertility depends on maintaining temperature stability during storage, banking, and transportation. Ref. [26] discovered that survival was considerably higher when queen banks were kept at 16 °C, above cluster temperature, as opposed to colder circumstances below cluster formation. Significantly, surviving queens from various thermal treatments had comparable fecundity and sperm viability, demonstrating that success mostly relied on keeping queens within a temperature range that avoids physiological disruption and cold-induced mortality. Their results validate the usefulness of above-cluster-temperature storage for long-term banking.

Because rising temperatures, more frequent heat waves, and unpredictable weather can affect stored sperm viability as well as the environmental conditions necessary for successful mating, climate change is becoming more widely acknowledged as a danger to queen reproductive resilience. In a comprehensive analysis of the effects of climate change on honey bees and beekeeping, Ref. [27] found that temperature and other weather factors typically have detrimental effects on a number of biological processes in *Apis mellifera*, such as metabolism, mortality, and colony function. Climate change is a significant abiotic factor for honey bee health, Ref. [28], who also noted that its effects combine with other stressors rather than acting alone. These climatic shifts are particularly significant for queen reproduction because long-term fertility depends on the thermal stability of sperm stored after mating, whereas successful mating depends on brief windows of ideal temperature, wind, and flying conditions. Even in cases where queens survive the exposure, direct evidence demonstrates that heat stress lowers queen fecundity. Ref. [29] discovered that short-term treatment of queens to cold shock decreased stored sperm viability. These results show that queens can experience hidden reproductive harm following heat stress and still appear normal on the outside.

Because queen fertility relies on healthy drones and successful mating flights, heat stress is also crucial before sperm storage. After reviewing the variables influencing drone reproductive health, Ref. [30] came to the conclusion that temperature stress can lower the quality and viability of drone sperm, which in turn lowers queen fertility. This implies that even if queens are not directly exposed to intense heat during mating, climate change may still have an adverse effect on queen reproduction. Additionally, the number of mating opportunities may change due to changes in the weather. Ref. [13] demonstrated that climate has a substantial impact on the frequency of queen mating, with rainfall having a negative correlation with polyandry in *Apis mellifera jemenitica*. According to their research, the frequency of successful matings is decreased by unstable or inappropriate flying weather, which limits the variety and amount of sperm queens can store. Any climate-related disruption to this window can have long-term effects on reproduction because mating takes place within a brief early life phase.

Climate change is therefore anticipated to have both behavioral and thermal effects on mating windows. Ref. [31] highlighted that controlled and favorable environmental circumstances are necessary for effective queen mating, and their revised queen-rearing standards take into account the significance of weather and timing during mating management.

The sensitivity of honey bee flight behavior to environmental factors and the significance of favorable weather for reproductive flight activity were also discussed by [32]. Mating windows may become shorter, less predictable, or more physiologically costly under more unpredictable climate circumstances, particularly when high temperatures are accompanied by poor floral conditions, wind, or precipitation. Ref. [27] showed that there is still a lack of research on the effects of climate change on honey bees at the apiary and beekeeper level. However, the evidence that is currently available suggests that changes in weather patterns can interfere with the temporal conditions required for normal reproductive performance. Collectively, these studies demonstrate that queen reproductive tissues are highly sensitive to thermal disruption across both commercial and environmental contexts. Importantly, sperm viability frequently declines before visible queen failure becomes apparent, suggesting that hidden thermal damage may contribute substantially to delayed colony decline.

### 3.2. Nutritional Stress

Carbohydrates for immediate energy and nutrients acquired from pollen for reproductive maintenance are the main sources of macronutrients during storage. According to [33], colonies that received both pollen substitute and sucrose solution had far higher rates of queen egg laying, larger enclosed brood areas, and larger honey reserves than colonies that did not get additional nourishment. Similarly, Ref. [34] found that queens reared in colonies fed sugar, honey, and fresh pollen had larger spermathecae, more ovarioles, and higher body weight, indicating that nutrient-rich diets improve reproductive quality. These findings are relevant to storage biology because banked queens depend on host-colony nutrition and attendant workers. Nutritional stress and reduced floral resources may therefore impair queen reproductive resilience by limiting egg production, sperm maintenance, and brood rearing. Ref. [35] highlighted the close relationship between queen egg laying and ambient nutrient supply. Because decreased diversity in nectar and pollen sources can diminish the amount and nutritional quality of nutrients reaching the queen, floral diversity loss is particularly critical to queen resilience.

There is now direct evidence that queen reproductive performance is impacted by floral abundance. In comparison to queens assessed at the start of flowering, Ref. [36] showed that queens raised and operating under floral shortage had worse emergence success, lower body weight, less oviposition, and inferior sperm quality in the spermatheca. Sperm concentration, motility, viability, acrosome integrity, mitochondrial activity, and seminal volume were all considerably enhanced by floral abundance, suggesting that seasonal resource scarcity impacts both the quality of stored sperm and current reproductive performance.

Ref. [37] demonstrated that commercial diet supplements frequently contained low linolenic acid and imbalanced omega-6:omega-3 ratios. They contended that these deficits may help clarify why artificial diets do not fully maintain colony health. Because queens rely on the nutritional quality of the pollen resources collected and processed by workers, this study’s findings are extremely pertinent to reproductive resilience, even if it did not specifically look at queens. Therefore, queen fertility is probably impacted by nutritional simplicity in damaged environments due to deficiencies in critical fatty acids and other pollen-derived nutrients that are difficult to replenish by poor forage or artificial diets.

By restricting access to pollen sources rich in vital proteins, lipids, and fatty acids needed for long-term queen fertility, landscape degradation and decreased floral diversity exacerbate nutritional stress [27]. Research has shown that nutritionally simplified settings degrade reproductive performance, decrease colony resilience, and hinder brood production. Pollinator resistance to other environmental stresses, such as exposure to agrochemicals, may also be influenced by resource quality [38]. Furthermore, the nutritional diversity of natural pollen is frequently not entirely replicated by artificial diets, especially when it comes to the balance of key fatty acids [37]. Together, these results suggest that long-term queen reproductive resilience depends on the maintenance of varied and consistent floral supplies.

### 3.3. Agrochemical Stress

By affecting sperm viability, ovarian function, pheromonal signaling, egg laying, and colony-level reproductive stability, pesticides and other agrochemicals can reduce queen reproductive resilience even at sublethal doses. One of the most compelling studies [39] showed that exposure to imidacloprid dramatically reduced sperm viability in the spermatheca and altered gene expression related to development, immunity, and antioxidation. Collectively, these findings demonstrate that sublethal pesticide exposure can directly compromise the queen’s stored reproductive reserve rather than merely affecting worker behavior or overall colony performance. Ref. [40] showed that the queens raised in wax with field-relevant levels of agrochemicals or miticides had smaller worker retinues, reduced egg-laying rates, and changed mandibular gland pheromone profiles. Additionally, their study revealed that workers reacted less strongly to queens exposed to pesticides, suggesting that developmental exposure to contaminated wax affects social signals that support queen function within the colony as well as reproductive performance. Because worker attention and care are just as vital to the queen’s success as her internal fertility, this type of reproductive toxicity is very significant.

According to other research, pesticide stress might indirectly hinder reproduction by raising the likelihood of queen replacement and upsetting colony reproductive function. Colonies fed pollen tainted with field-relevant chemical mixes showed higher brood loss and, under the fungicide treatment, higher queen loss, according to [41]. This study shows that sublethal agrochemical exposure can compromise the colony’s reproductive continuity through impacts on both brood survival and queen persistence, even though it focused on colony-level outcomes rather than isolated queen physiology. Additionally, Ref. [42] demonstrated that indirect exposure to insect growth disruptors changed ovarian protein expression and queen reproductive behavior, with some treatments decreasing daily egg laying. According to their findings, queen reproductive physiology can be impacted by agrochemicals even when exposure takes place through the social context as opposed to direct dosing.

Sperm viability alone may not necessarily show the damaging effects of pesticides on queen resilience, and some substances cause more subtle molecular disturbance. While sperm viability and queen longevity were not significantly decreased under their field conditions, Ref. [43] discovered that sublethal amitraz exposure changed queen gene expression linked to detoxification, cAMP-dependent protein kinase, immunity, antioxidant capacity, and development. These findings suggest that certain agrochemicals can generate cryptic physiological stress without causing immediate reproductive collapse, potentially contributing to delayed or difficult-to-diagnose queen failure.

Recent research confirms that ovarian and metabolic pathways can be involved in reproductive damage. According to [44], queens exposed to the fungicide boscalid experienced disruptions in colony dynamics and reproduction, including a decrease in the quantity of sperm stored in the spermatheca and a decrease in the expression of vitellogenin. These effects were similar to nutritional stress, suggesting that fungicides can hinder reproduction through both direct toxicity and disturbance of the metabolic processes necessary for long-term egg production and fertility maintenance. Together, these findings expand the concept of pesticide-related reproductive toxicity beyond insecticides alone and demonstrate that fungicides may also impair queen reproductive function through metabolic disruption.

### 3.4. Pathogen and Parasite Stress

Ref. [45] showed that varroosis predominates in evidence-based beekeeping research, indicating its crucial role in colony decline and management. *Varroa destructor* mostly parasitizes workers and brood, but its effects on reproduction are very important for queens because mite-driven viral pressure lowers colony stability, raises the pathogen load surrounding the queen, and causes queen failure and supersedure. In commercial settings, where queen resilience depends on a steady nurse-worker environment and low infectious load, this indirect approach is particularly crucial.

*Nosema ceranae* further compromises reproductive resilience by inducing chronic physiological stress and intensifying the effects of other environmental stressors. Ref. [46] demonstrated that semen quality was significantly impacted by infection and that *Nosema ceranae* alone caused quantifiable physiological impairment in drones. More significantly, drone physiology and survival significantly decreased when *Nosema ceranae* was mixed with the pesticide fipronil. The authors came to the conclusion that such impairment might decrease the success of mating and cause queens to store less diverse and high-quality semen. Because a queen’s long-term fertility depends on the quality of semen obtained during mating, pathogen pressure on drones can directly result in impaired queen performance, making this extremely pertinent to queen reproductive resilience. The idea that *Nosema ceranae* is especially harmful when it acts in concert rather than alone is supported by [28], who also highlighted how a variety of interacting biotic and abiotic factors influence honey bee health.

Viruses represent one of the clearest direct biological threats to queen reproductive function because infection is associated with reduced ovarian development, impaired egg laying, and increased supersedure. According to [47], common viral infections are linked to early supersedure in colonies, limit egg laying in honey bee queens, and lower ovarian size. While field observations verified that increased viral infection indicated the existence of supersedure cells, their laboratory studies revealed that queens infected with live virus had smaller ovaries and were less likely to resume egg laying than controls. These findings offer compelling proof that the traditional signs of queen failure are closely associated with viral infection. Similarly, Ref. [48] discovered that experimental infection with Israeli acute paralysis virus directly reduced ovary size and decreased vitellogenin expression, while natural viral infection was linked to decreased ovary mass, lower sperm viability, and altered ovary protein composition in queens. Collectively, these studies demonstrate that viruses affect sperm-related reproductive parameters and ovarian function in queens.

The way viruses interact with immunity and other stressors increases their significance. In their analysis of viral co-infections and antiviral immunity in honey bees, Ref. [49] highlighted that the effects of infection depend on interactions between viruses and the host immune system in addition to the virus itself. The idea that co-infection can worsen pathogenic outcomes and make recovery more difficult is supported by their review. After reviewing honey bee queens and viral infections in further detail, Ref. [50] came to the conclusion that viruses can impact queen reproductive vigor through both vertical and horizontal transmission channels. In the context of reproductive resilience, this is particularly crucial because queens may serve as generational nodes of disease transmission in addition to being exposed to the surrounding colony environment. Together, these studies demonstrate that pathogen-related reproductive decline operates through interconnected effects on immunity, ovarian physiology, sperm quality, and colony-level social stability.

Therefore, the key to comprehending pathogen-related reproductive decline is synergistic stress. *Nosema ceranae* and a pesticide can interact to cause more severe reproductive damage than either stressor alone, according to direct data presented by [46]. Pesticide treatment decreased sperm viability and changed immunological and detoxification gene expression in queens, as demonstrated by [39]. This suggests that chemical stress and disease vulnerability can coexist in the same reproductive systems. According to [28], colony losses are challenging to explain using single-factor models due in large part to the interactions between parasites, diseases, pesticides, and management techniques. Collectively, these findings indicate that pathogen-related reproductive decline rarely occurs through a single mechanism. Instead, viruses, parasites, and microsporidian infections frequently interact with nutritional, environmental, and management stressors to weaken queen fertility and long-term colony resilience. Importantly, the reviewed evidence suggests that reproductive failure in queens is most severe when pathogen pressure interacts with nutritional limitation, pesticide exposure, or thermal stress, highlighting the multifactorial nature of queen decline under commercial conditions.

Every stressor has a different effect depending on when it happens in the queen’s life. Major stressors are mapped onto the particular stages of development and reproduction that they interfere with in Table 1.

### 3.5. Commercial Handling, Banking, and Transport Stress

Although queen banking is a common commercial practice, it is not physiologically neutral for queens. In their description of queen handling and banking as a standard part of contemporary queen raising and commercial breeding, Ref. [31] emphasized that stored queens need to be carefully managed because sealing and delayed introduction can affect subsequent quality. Ref. [56] found that the banking environment changed the composition of the gut microbiota, oxidative stress-related gene expression, and microbial succession in queens retained in active colonies as opposed to queen banks. According to [56], queen banks’ low-metabolic, less social milieu deviates significantly from typical colony assimilation and is linked to physiological profiles that are inconsistent with maximum reproductive activity.

Density of storage is also important. In their evaluation of queen banks with 40 or 80 queens, Ref. [10] discovered that while lower-density banking produced better results, increasing bank density significantly decreased queen survivability during bank storage. Compared to queens that overwintered freely in colonies, queens retrieved from bank colonies were initially lighter and smaller. During the initial weeks following introduction, their oviposition decreased, but their sperm viability and count remained unchanged. These results suggest that while queen banking can maintain reproductive potential, there are still short-term functional consequences upon release. Publicly available research on the storage of virgin queens also demonstrates how storage conditions within colonies affect survival and acceptance later on. Virgin queens kept in cages within queenless colonies fared better than those kept in queenright colonies, according to [60], demonstrating that the host colony’s social context has a direct impact on caged queen maintenance. In a similar study, Ref. [61] showed that later supersedure rate was influenced by queen density, cage level, and cage position inside queenright bank colonies.

**Figure 2:**
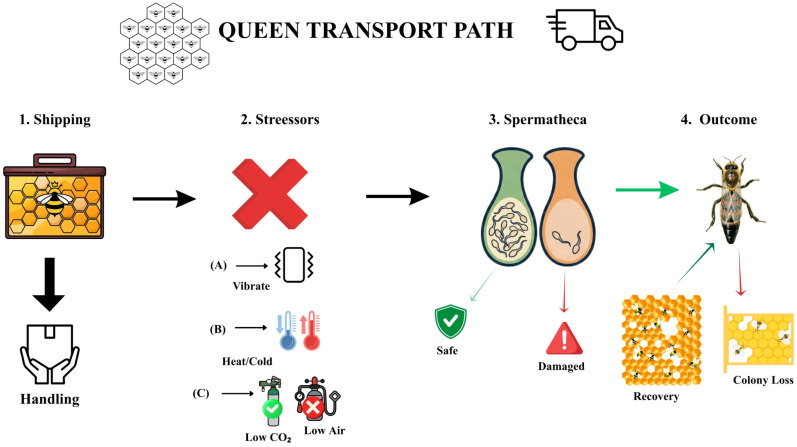
Computational model of the physiological effects of queen bee transit stress. (A) Vibration, (B) Heat/Cold exposure, and (C) Low CO_2_/Low Air represent the fluctuating environmental variables queens encounter during transit, such as temperature spikes, vibration, and motion. This example shows how honey bee queens are subjected to multifactorial stress due to fluctuating commercial transit conditions, which immediately damages stored sperm and causes delayed queen care and connected shipment-related stress to subsequent colony performance.

Commercial transit exposes queens to stressful conditions that lower reproductive quality, according to the most convincing data from shipment studies. According to [5], queens from failing colonies had significantly lower sperm viability than queens from healthy colonies, and queens exported under commercial settings experienced temperature spikes. Their research demonstrated that shipping is not a neutral step in queen care and connected shipment-related stress to subsequent colony performance, as shown in Figure 2.

Additionally, Ref. [9] tracked queen shipments and showed that while box design and the presence of attendant bees affect the degree of protection during transport, queens encounter varying travel conditions. Collectively, these findings indicate that commercial queen management is not physiologically neutral. Banking density, confinement conditions, transport design, and prolonged handling can all influence queen reproductive performance, even when queens survive and appear externally healthy. The major stressors affecting queen reproductive resilience, their principal biological targets, and their reproductive consequences are comparatively summarized in Table 2.

## 4. Emerging Technologies, Knowledge Gaps, and Future Directions

### 4.1. Instrumental Insemination and Reproductive Technologies

**Figure 3:**
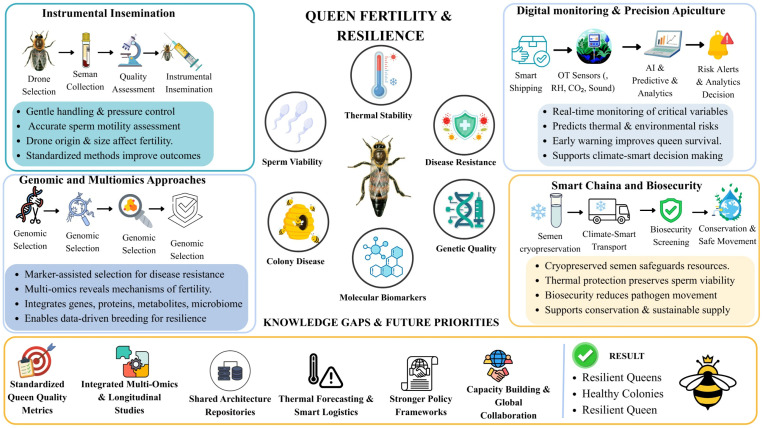
Emerging technologies and future directions for the resilience and fertility of honey bee queens. An outline of significant developments in cryopreservation, climate-smart supply chains, biosecurity, genomic and multi-omics techniques, digital monitoring and precision apiculture, and instrumental insemination. Improved sperm viability, temperature stability, disease resistance, genetic purity, colony performance, and sustainable apiculture are all supported by these integrated technologies, as the figure illustrates.

Sperm viability decreases both during the second stage of endophallus eversion and during the injection of semen into the queen’s lateral oviducts, as demonstrated by [62]. Their research provided a clear technical explanation for why the results of instrumental insemination can differ by showing that elevated pressure during semen preparation and insemination is a primary source of sperm destruction. This finding has practical significance since it changed focus to more cautious ejaculate manipulation, pressure management, and gentle semen handling during insemination techniques (Figure 3).

Technical and biological factors both influence the quality of semen used in instrumental insemination. Measured sperm motility can vary depending on chamber design, diluent composition, incubation conditions, and analytical methods [63]. In addition, drone rearing environment and subspecies background affect sperm concentration, ATP content, semen volume, and ejaculation efficiency [64,65].

Ref. [66] demonstrated that semen from larger drones produced higher paternity shares by instrumentally inseminating queens with varying quantities of semen from large and small drones. These results are pertinent to technological fertility control because they demonstrate that post-insemination sperm competition dynamics within the queen have an impact on insemination success in addition to semen quantity and viability.

These technological advancements have been integrated into practical breeding practice through recent standardization initiatives. Instrumental insemination is a crucial technique for controlled mating in queen breeding, according to [31], who also highlighted its function in preserving specific features, bolstering pedigree accuracy, and enhancing quality control in contemporary breeding programs. This is reflected in their updated standard techniques. The usefulness of this approach in breeding programs was confirmed by [67], who also demonstrated that colonies led by instrumentally inseminated queens can successfully express certain breeding traits, with performance outcomes comparable to naturally mated queens in numerous respects.

Long-term germplasm management and ex situ conservation are further applications of instrumental insemination. In their study of the increasing significance of drone semen storage as a supplement to breeding and conservation techniques, Ref. [68] emphasized the necessity of instrumental insemination for using preserved semen in genetic resource management. Collectively, these studies demonstrate that reproductive technologies are becoming increasingly important not only for controlled breeding but also for long-term genetic conservation and resilience management in commercial apiculture.

### 4.2. Digital Monitoring and Precision Apiculture

Direct transport studies indicate the necessity of predictive monitoring during queen transportation. According to [9], sperm viability in the spermatheca is greatly reduced by exposure to both low and high temperatures, and queens are subjected to varying thermal conditions during transportation. Additionally, they discovered that the thermal buffering of queens during transit is influenced by the form of shipping boxes and the presence of attendant bees. Transport circumstances can have long-lasting effects on reproduction, as demonstrated by [5], who also connected shipment-related temperature spikes to decreased sperm viability and colony failure. These studies demonstrate that in-transit circumstances are quantifiable risk factors, making them appropriate targets for sensor-based monitoring.

Continuous sensing and automated prediction are already possible in apiculture more generally, according to published research on precision beekeeping. In a thorough study of precision beekeeping, Ref. [69] showed that contemporary monitoring systems increasingly combine exterior environmental factors with inside-hive characteristics, such as weight, temperature, humidity, sounds, vibrations, and gases. Their analysis demonstrates that sensor-based monitoring is now a recognized area of beekeeping research and offers the technical framework for expanding these techniques to queen transportation systems. These advances suggest that precision-monitoring systems may eventually allow earlier detection of hidden reproductive stress before measurable colony decline becomes visible under field conditions.

### 4.3. Genomic and Multi-Omics Approaches for Resilience

Phenotypic breeding has given way to molecular and marker-assisted methods in the selection process for disease resistance. Selecting resistant lineages is one of the most sustainable alternatives to frequent acaricide use, according to [70], who reviewed resistance breeding against *Varroa destructor*. However, success depends on selecting biologically relevant traits and minimizing environmental distortion of trait expression. According to their study, breeding programs can incorporate resistant traits such hygienic behavior, Varroa-sensitive hygiene, and inhibition of mite reproduction; nevertheless, field deployment requires improved trait validation and uniformity. Strong field evidence that long-term selection under commercial conditions may produce colonies with consistently low mite populations and survival without treatment was presented by [71], demonstrating that disease-resilient breeding is practical rather than merely theoretical.

The incorporation of resilience qualities into queen and colony improvement is also supported by broader breeding frameworks. According to [31], disease resistance is still one of the most crucial aims in contemporary queen selection programs, which increasingly rely on molecular techniques, pedigree integration, and standardized performance testing.

Additionally, Ref. [72] shown that sperm heat resilience and drone survival under heat challenge differ by population origin, with Southern Californian drones exhibiting higher heat resilience than Northern Californian drones. Because drone thermal tolerance directly impacts queen fertility, their findings suggest that heat resilience has population-level variation and thus potential breeding implications. Ref. [73] also demonstrated that quick heat hardening of queen larvae changed antioxidant and stress–response patterns in daughter workers, bolstering the notion that developmental conditioning can affect thermal resilience and may have physiological effects that span generations.

Nevertheless, compared to disease-resistance breeding, direct genomic selection for heat tolerance in honey bees is still in its early stages. In their analysis of thermal adaptations in social insects, Refs. [74,75] highlighted the importance of behavioral, physiological, and molecular responses at the individual and colony levels for temperature tolerance. This suggests that rather than being a single, easily selectable signal, thermotolerance in honey bees is rather a complex characteristic altered by several pathways. Although evidence suggests heritable and population-level variation in heat resilience, current breeding programs still focus mainly on disease and parasite resistance. A major limitation in honey bee queen research is the lack of standardized quality measures across breeding, storage, transportation, and post-introduction studies. Queen quality is multifaceted rather than reducible to a single indicator, as showed by [2], who reported that commercially produced queens differ significantly in physical characteristics, insemination success, and reproductive condition, including sperm count, sperm viability, and queen size. By updating standard procedures for queen rearing, handling, selection, and performance testing, Ref. [31] explicitly addressed this issue, highlighting the need for harmonized procedures for queen production, mating control, and colony-level evaluation in order to achieve comparable quality assessment. Their work demonstrates that if results from many breeding programs, commercial systems, and experimental investigations are to be evaluated collectively, methodological standardization is still crucial.

Additionally, published biomarker studies demonstrate the fragmented nature of existing queen-quality assessment. In addition to identifying spermathecal proteins linked to both sperm viability and queen failure, Ref. [19] suggested potential stress biomarkers for the diagnosis of queen failure in field settings. These findings show that molecular markers can identify cryptic reproductive decline that is not always reflected in outward characteristics like queen survival or brood pattern. Ref. [76] pointed out that while proteomics has already shown potential biomarkers for queen condition and honey bee health, these markers are not yet incorporated into a widely accepted quality framework. Because there is not a single established standard that unifies them, researchers and beekeepers continue to rely on partially overlapping measurements, such as morphometrics, sperm viability, ovary size, laying pattern, and molecular fingerprints.

Because different stressors impact different aspects of queen quality, this lack of consistency is particularly troublesome. While [55] discovered that queens may seem phenotypically normal following thermal stress even when sperm viability has diminished, Ref. [9] demonstrated that temperature extremes during transportation reduce sperm vitality. While [44] showed that fungicide exposure decreases stored sperm and vitellogenin expression, Ref. [47] revealed that viral infection decreases ovarian size and egg laying and is associated with premature supersedure. According to these studies, there isn’t a single indicator that can accurately describe queen quality in every situation. Therefore, rather than depending solely on individual features, future research needs standardized diagnostic panels that incorporate structural, reproductive, physiological, and colony-response parameters.

#### 4.3.1. Transcriptomics and Proteomics of Queen Fertility

The integration of several omics layers is a second important research priority. Important but largely distinct insights into queen fertility and stress response have already been revealed by current omics-based research. Ref. [17] identified genes in the spermatheca linked to sperm storage following mating using transcriptomics. Ref. [72] used proteomics to demonstrate how mating changes the proteome of spermathecal fluid, including proteins involved in glucose metabolism and antioxidants. Long-term sperm preservation in the spermatheca is linked to distinctive lipid and metabolic patterns, as demonstrated by [18] using metabolomics. Ref. [24] further showed the biological connection between microbiome composition, metabolism, and reproductive function by connecting alterations in gut microbiota with ovarian metabolism in queens. These findings collectively demonstrate that transcriptomic, proteomic, metabolomic, and microbiological levels all affect fertility and resilience, although these levels are still far too frequently investigated separately.

#### 4.3.2. Multi-Omics Integration and Systems Biology

As increasing research demonstrates that different molecular layers do not necessarily convey the same information, the need of multi-omics integration has become more evident. Ref. [77] combined transcriptomics, proteomics, and microbiome profiling in an integrated honey bee study outside of the queen-specific context and discovered that microbiome composition linked with metabolic modules and that tissue-specific protein networks differed from RNA-based networks. Their findings show how important systems-level research is for finding biologically significant correlations that single-omics methods would overlook. Additionally, Ref. [78] stressed that modern standard procedures, interoperable pipelines, and best practices in genomics, proteomics, and microbiome sampling are now necessary for honey bee omics research. This is especially crucial for studies on queens, since the reproductive phenotype depends on the synchronization of tissues, proteins, metabolites, gene regulation, and microbial ecosystems.

#### 4.3.3. Future Needs and Standardization

The advantages of such integration are already hinted at in published queen studies. Ref. [24] demonstrated the interconnected effects of reproductive suppression on gut microbiota, ovarian gene expression, and glycerophospholipid metabolism by combining microbiome analysis with ovarian transcriptome and metabolic data. Hindgut symbiont abundance was connected by [25] to significant host transcriptome changes related to queen age and quality. According to [79], viral infection in queens modifies molecular profiles, reproductive characteristics, and pheromone production. According to these findings, queen failure is a systems-level trait rather than a defect in a specific organ or molecule. Thus, longitudinal multi-omics studies that track queens during rearing, mating, storage, shipping, introduction, and colony establishment while integrating physiological and colony-level outcomes should be a priority for future study. The absence of a data architecture for combining queen-related omics and phenotypic data is another significant gap. While [78] emphasized the necessity of updated standards and interoperable techniques in honey bee omics, Ref. [74] emphasized the significance of accessible and reusable proteome archives. Even high-quality omics data are difficult to compare between studies without comparable metadata on queen age, breeder origin, mating status, storage circumstances, transport history, pathogen burden, and colony performance. Therefore, standardization of molecular operations and standardization of quality measurements must progress together. Future integration of physiological, molecular, microbial, and colony-level datasets will likely be essential for developing predictive frameworks capable of identifying reproductive decline before irreversible colony damage occurs.

### 4.4. Cryopreservation, Climate-Resilient Supply Chains, and Biosecurity

Whether the present developments in cryobiology can be translated into dependable, field-relevant reproductive preservation will be crucial to the future of queen fertility management. Instead of whole-queen preservation, drone semen cryopreservation has yielded the most significant advances thus far. Honey bee semen reacts to controlled cryobiological design similarly to other animal germplasm systems, as shown by [80], who reported that systematic tuning of diluents, cryoprotectants, and semen dilution ratios could significantly improve post-thaw sperm vitality. In order to demonstrate that frozen germplasm may enable the production of fertile female progeny and therefore serve as a real conservation and breeding resource, Ref. [81] produced several generations of queens using cryopreserved semen. This foundation has been improved by more recent work. Ex situ preservation of drone germplasm is one of the most promising methods for long-term conservation of *Apis mellifera* genetic resources, according to [82], who examined both cryopreservation and above-freezing semen storage solutions. However, sperm quality after thawing and the fertility of inseminated queens continue to be the main performance limitations. Drone semen cryopreservation, according to [68], should be viewed as a crucial supplement to in situ conservation initiatives since it enables the preservation of valuable lines with characteristics like environmental adaptation, hygienic behavior, and winter hardiness.

However, published data indicates that significant structural and functional damage to sperm still limits cryopreservation, making further technical improvement a top research goal. Long-term cryopreservation alters drone sperm ultrastructure, including axoneme vacuolation, intranuclear vacuoles, and mitochondrial loss, as demonstrated by [57]. In their comparison of glycerol, dimethyl sulfoxide, and trehalose as cryoprotectants, Ref. [83] emphasized the ongoing need to adjust the composition of cryomedia. More recently, Ref. [59] found that queens inseminated with treated sperm had more motile sperm in the spermathecae, and that cryoprotectant-free vitrification using royal jelly enhanced post-warming sperm motility, viability, and DNA integrity. Ref. [84] also demonstrated that while cryopreserved semen continued to perform worse than fresh semen overall, a straightforward antibiotic-free freezing procedure could nevertheless sustain queen survival to egg laying and female brood generation.

According to [5], a significant percentage of stored sperm can be killed when queens in commercial shipments are exposed to temperatures below 8 °C and above 40 °C. In addition to confirming that shipping conditions have a significant impact on the quality of queen sperm, Ref. [9] showed how box design and attendant bees affect the thermal buffering of queens throughout transit. According to [55], even when stored sperm viability has already decreased, queens can still seem outwardly normal following brief temperature stress. All of these results point to the necessity of designing climate-smart queen supply networks with sperm thermal protection in mind rather than only the queen’s survival.

Climate-smart supply chains must account for the increased frequency of heat shocks during production and transport driven by climate change. In their analysis of the effects of climate change on honey bees, Ref. [27] highlighted the growing threat to beekeeping systems posed by rising temperatures and unpredictable weather.

This future priority also includes the creation of more intelligent monitoring systems. Precision beekeeping today frequently incorporates remote sensing of temperature, humidity, weight, sound, and other colony factors, as demonstrated by [69,85]. These technologies offer a clear technological foundation for climate-smart transport management, even though they are not yet tailored to individual cargo. The biological factors that are important during shipment, particularly thermal exposure, have already been discovered by [5,9], and precision-apiculture experiments demonstrate the growing viability of IoT-based monitoring and predictive analytics. Therefore, translating these technologies from colony-level monitoring into queen-specific supply chains that can identify high-risk transit events before reproductive damage accumulates is the top research objective.

Because queen mobility is both economically essential and biologically hazardous, policy and biosecurity considerations are equally significant. In their assessment of the migration of western honey bees across the United States, Ref. [7] showed that while moving live bees and queens promotes pollination and breeding, it also raises the danger of pests, diseases, and uneven regulation. Their research demonstrates that bee movement biosecurity frameworks are still inconsistent and that improved coordination of inspection, permission, and mitigation procedures is necessary for safer movement. Because queens play a key role in the exchange of germplasm, breeding, and colony replacement, this directly relates to queen supply chains.

Because reproductive material itself can harbor infections, biosecurity issues are heightened. Ref. [86] found viral sequences in drone semen and offered proof that viruses might spread vertically using drones. Because it demonstrates that reproductive technologies can also become pathways of pathogen migration if biosecurity screening is insufficient, this finding is especially significant for semen-based breeding and artificial insemination. The necessity of standardized techniques for queen breeding and selection was highlighted by [31]. This implicitly entails more stringent control over handling, origin, and quality evaluation. Ref. [68] further contended that ex situ conservation through semen preservation should supplement rather than replace more comprehensive management measures, arguing that disease-risk management and genetic resource protection need to be connected. Therefore, future policy must include semen, queens, and breeding stock as possible biosecurity vectors as well as valuable reproductive material.

Another policy concern is that improper management of queen mobility may lead to conflicts between breeding and conservation objectives. According to [81], cryopreserved semen can help maintain or restore honey bee lines, providing a way to conserve genetic variation without the need for frequent live translocation. The significance of semen preservation technology for maintaining regional germplasm was also emphasized by [82]. According to these studies, ex situ germplasm banks are becoming more and more supported by policy as a biosecure substitute for the regular long-distance transportation of live queens. These strategies could lessen the need for frequent shipping, minimize fertility losses caused by transportation, and support the preservation of locally adapted stocks.

## 5. Conclusions

Honey bee queen fertility is central to colony productivity, population stability, and long-term survival. This review highlights that queen reproductive resilience is shaped by interacting developmental, physiological, molecular, environmental, and management-related factors. Successful queen performance depends on effective mating, sperm viability and storage in the spermatheca, hormonal and metabolic balance, adequate nutrition, and favorable environmental conditions. Current evidence shows that queen fertility is highly vulnerable to multiple stressors. Thermal stress during storage, transport, and heat events can reduce sperm viability and impair long-term reproductive success. Nutritional stress, landscape degradation, agrochemicals, pathogens, and parasites may further compromise ovarian function, immune responses, pheromonal signaling, and colony stability. These stressors often act together rather than independently, creating hidden reproductive damage that may later appear as queen failure or colony decline. Commercial queen production practices, including queen banking, confinement, handling, and long-distance transportation, can also affect queen physiology, microbiome composition, and reproductive quality, even when queens appear externally healthy. Therefore, these practices should be considered important components of queen reproductive health management. Emerging tools such as precision apiculture, cryopreservation, instrumental insemination, and omics-based biomarkers offer promising opportunities to improve queen breeding, conservation, and stress detection. Future research should prioritize longitudinal studies that follow queens from development through mating, storage, transport, and colony establishment. Greater attention is also needed to link molecular biomarkers with colony-level outcomes and to develop standardized queen-quality assessment methods. Improving queen reproductive resilience will require better nutrition, stronger biosecurity, optimized storage and transport conditions, thermal protection, and selective breeding for stress and disease resistance. Ultimately, protecting queen fertility is an agricultural and ecological priority that requires collaboration among researchers, breeders, beekeepers, policymakers, and commercial queen producers.

## Figures and Tables

**Table 1:** Vulnerability Windows: Major Stressors Affecting Queen Fertility Across Life Stages.

Life Stage/Mechanism	Primary Stressors	Consequence of Disruption	Evidence
Larval development	Poor nutrition; pesticide exposure; poor colony conditions	Reduced ovariole number; smaller queens; decreased lifetime fecundity	[20,34,40]
Pupal development	Temperature stress; poor nutrition; miticide residues	Small or malformed spermatheca; reduced sperm storage capacity	[51,52]
Mating period	Adverse weather; limited drones; pesticides; poor drone health	Low sperm count; poor genetic diversity; failed post-mating activation	[13,30,39]
Post-mating activation	CO_2_ narcosis; handling stress; poor nutrition; viral infection	Failed sperm storage initiation; poor long-term fertility	[47,53,54]
Sperm storage & maintenance	Heat/cold stress; temperature fluctuations; pesticide exposure	Progressive sperm viability loss; premature queen failure	[5,9,55]
Sperm storage & maintenance	Viral infection (DWV, IAPV, ABPV); *Varroa destructor*	Immune-mediated sperm damage; vertical pathogen transmission	[47,50]
Ovarian function & egg laying	Nutritional scarcity; fungicide exposure; *Nosema ceranae*	Reduced egg laying; poor brood pattern; supersedure	[33,44,46]
Queen banking & transport	High-density banking; temperature spikes; poor ventilation	Sperm damage; temporary oviposition suppression; microbiome dysbiosis	[9,10,56]
Cryopreservation (semen)	Freeze–thaw cycles; cryoprotectant toxicity	Reduced post-thaw motility; structural sperm damage	[57,58,59]

**Table 2:** Comparative Summary of Major Stressors Affecting Honey Bee Queen Fertility and Reproductive Resilience.

Stressor	Primary Biological Impact	Major Reproductive Consequence	Representative References
Thermal stress	Sperm damage; heat-shock response	Reduced sperm viability; premature queen failure	[5]
Nutritional stress	Impaired metabolic and ovarian support	Reduced egg laying and reproductive quality	[33]
Agrochemical exposure	Oxidative stress; endocrine and immune disruption	Altered pheromonal signaling; reduced fertility	[39]
Pathogens and parasites	Viral infection; immune activation; colony pathogen pressure	Ovarian degeneration; supersedure; reduced fertility	[45]
Commercial handling and transport	Confinement stress; altered microbiota; shipping stress	Reduced oviposition; hidden reproductive damage	[9]
Climate and landscape stress	Floral-resource limitation; thermal instability	Reduced mating success and colony resilience	[13]

## Data Availability

No new data were created or analyzed in this study. Data sharing is not applicable to this article.

## Abbreviations

The following abbreviations are used in this manuscript:

20E	20-Hydroxyecdysone
ABPV	Acute Bee Paralysis Virus
AI	Artificial Intelligence
ATP	Adenosine Triphosphate
BQCV	Black Queen Cell Virus
CO_2_	Carbon Dioxide
cAMP	Cyclic Adenosine Monophosphate
DEGs	Differentially Expressed Genes
DMSO	Dimethyl Sulfoxide
DNA	Deoxyribonucleic Acid
DWV	Deformed Wing Virus
FV	Filamentous Virus
GAPDH	Glyceraldehyde-3-Phosphate Dehydrogenase
GPx	Glutathione Peroxidase
HSP	Heat-Shock Protein
IAPV	Israeli Acute Paralysis Virus
IoT	Internet of Things
JH	Juvenile Hormone
KEGG	Kyoto Encyclopedia of Genes and Genomes
LoRaWAN	Long Range Wide Area Network
MnSOD	Manganese Superoxide Dismutase
mTOR	Mammalian Target of Rapamycin
OBP	Odorant-Binding Protein
O_2_	Oxygen
RNA	Ribonucleic Acid
ROS	Reactive Oxygen Species
SBV	Sacbrood Virus
Vg	Vitellogenin
